# Maxillary Defect Rehabilitation Using a Hollow Bulb Obturator

**DOI:** 10.7759/cureus.31326

**Published:** 2022-11-10

**Authors:** Parthasarathy N, Anusha KS, Madhan Kumar S, Shanmuganathan Natarajan

**Affiliations:** 1 Prosthodontics, Sri Ramachandra Institute of Higher Education and Research, Chennai, IND

**Keywords:** rehabilitation, obturator, prosthesis, hollow bulb, magnets

## Abstract

Rehabilitation of maxillofacial defects is an arduous task that involves restoring the form and function of the resected part of the defect. Obturators are the preferred choice of treatment for patients after resection involving the maxilla. Rehabilitation aims to separate the oral and nasal cavity and maintain anatomy while also assisting the patient with mastication, maintaining occlusion, supporting the mandible and facial tissues, and reestablishing speech and esthetics. Following surgery, a prosthetic evaluation is performed in which magnets are attached to the prosthesis to restore function and speech, as well as retention, stability, and quality of life. This case report describes how a hollow bulb obturator was used to treat a partial maxillectomy patient with magnets and clasps for patient rehabilitation.

## Introduction

Oral maxillofacial defects can be congenital or acquired due to trauma or pathology [1]. Rehabilitation of these defects is difficult because it necessitates a multidisciplinary approach that includes surgical, prosthodontic, psychological counseling, and speech therapy rehabilitation for the patient’s overall well-being [1]. Maxillofacial defects caused by neoplasm necessitate surgical resection of the nasal, maxillary, and oral cavities [1]. This results in communication between the oral-nasal cavity and the antrum. The rehabilitation of this defect entails precise compartment separation, which prevents nasal cavity regurgitation, prenasal speech, and defective function of the maxillary space resected surgically [1]. The goal of the rehabilitation is to reinstate speech, mastication, and occlusion, to prevent enophthalmos and diplopia, to focus on providing soft tissue support to restore the midfacial contour, and to achieve an adequate esthetic result that enhances both the patient’s physical and psychological well-being [2].

Ambroise Paré pioneered the artificial closure of palatal defects in the 1500s, and Martin spearheaded the fabrication of surgical obturators in 1875 [2]. Fry used prosthodontic impressions to close surgical defects after surgery in 1927, and Steadman used gutta-percha to stabilize an acrylic resin prosthesis on a skin graft to seal a maxillectomy defect in 1956 [2]. Surgery to close the defect is not recommended due to the extent of the deformity, where access is limited, and vascularization, as well as in some instances where recurrence of the lesion is suspected depending on the intensity of the tumor [1]. Obturators are used to rehabilitate maxillofacial defects by providing temporary obturators to allow surgically operated tissues to heal before prosthetic reconstruction [2]. Obturators provide a stable matrix after surgery, reducing the psychological impact of surgery, preventing nasal regurgitation, and allowing effective speech after surgery while also improving esthetics [3]. Meatal obturators are used to repair large soft palate defects. Although prosthesis extends where there is no muscle activity, these obturators cause changes in nasal resonance and air emission, which cannot be controlled [4].

The extent of the obturator varies depending on the type of defect. Aramany’s classification of maxillary defects is commonly used as follows: Class I, a defect where the resection is performed in the anterior midline of the maxilla, with abutment teeth present on one side of the arch; Class II, a defect that is unilateral, retaining the anterior teeth on the contralateral side; Class III, a palatal defect that occurs in the central portion of the hard palate and may extend into the soft palate; Class IV, a defect that crosses the midline and involves both sides of the maxilla, with abutment teeth present on one side; Class V, a surgical defect that is bilateral and lies posterior to the abutment teeth (labial stabilization may be needed); and Class VI, an anterior maxillary defect with abutment teeth present bilaterally in the posterior segment [5]. This case report is classified as Aramany’s Class I defect. When making a prosthesis, it should be light in weight, stable, retentive, and comfortable for the patient [6]. Obturator fabrication techniques, both open and closed hollow bulb obturators, rely on providing patient comfort, ease of use, and full coverage of the defect [6]. Open bulb obturators are simple to maintain, but the main disadvantage is mucus accumulation, which necessitates the placement of vents in the hollow section to prevent mucus, food, and fluid accumulation, which leads to foul odor and taste [7]. Closed bulb obturators extend to the superior extent of the defect and reduce air space and mucus accumulation, reducing the weight of the prosthesis by around 30%-35%, which is an added benefit in these prostheses [7]. A lightweight prosthesis was created in the literature using various techniques and materials. Wax shim, gelatin soap, cellophane wrapped in asbestos, polyurethane foam, plaster of Paris and pumice slurry, acrylic resin shim, modeling clay, dental stone, sugar, salt, alum, silicone putty, light-body coated gauze, thermocol, and iceblock are used to create a hollow in the prosthesis and reduce the weight of the prosthesis [8]. This article describes the indigenous technique of fabrication of a two-piece complete hollow bulb obturator for a patient with subtotal maxillectomy.

## Case presentation

Following the surgical removal of squamous cell carcinoma, the Department of Oral Surgery referred a 40-year-old male patient to the Department of Prosthodontics for rehabilitation. Just after a maxillectomy on the right side, the patient had a collapsed midface. A significant portion of the nasal septum, part of the inferior nasal conchae, until the antrum of the right side with teeth 22-28 present on the left side was surgically excised. As the tissues required healing, a surgical obturator was used for a month, followed by an interim obturator to help the patient resume his normal social life [2]. He was then called back for the fabrication of a definitive obturator. In this case, a two-piece hollow bulb obturator was made using the indigenous technique. The patient was informed of the procedure, and consent was obtained before the impression procedure.

**Figure 1:**
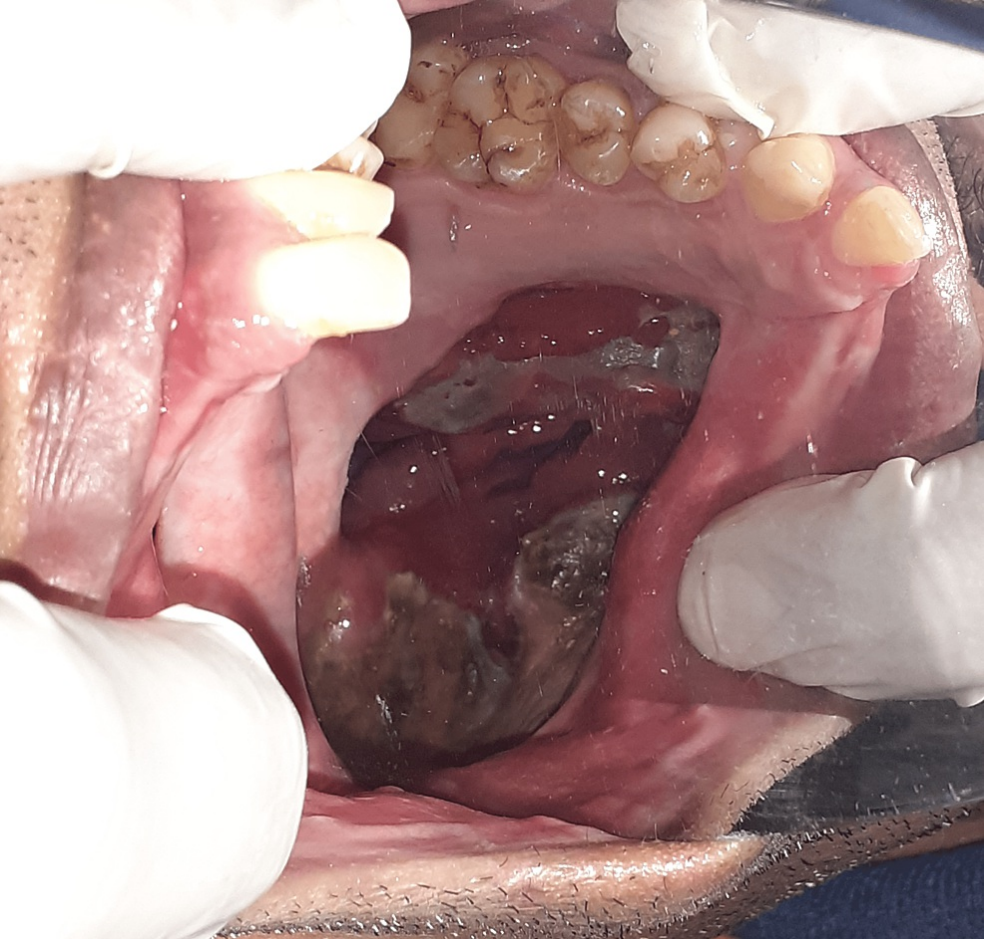
Site of the defect

Before the impression procedures in the patient, the site of the defect area, excluding the retentive undercut regions, was carefully isolated with gauze coated with petroleum jelly to prevent the impression material from being locked into the undercuts after the material was set (Figure 1). The defect was recorded using a putty viscosity addition silicone impression material (Zhermack Elite, Vasa Denticity, New Delhi, India), and dental stone (Kalstone, Vijai Dental, Chennai, India) was used for pouring the cast of the bulb portion.

**Figure 2:**
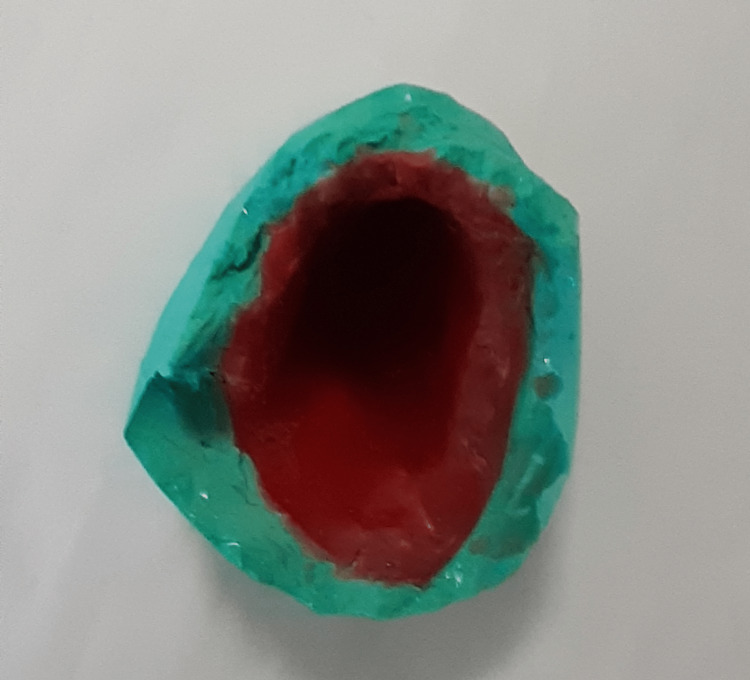
Bulb lined with modeling wax

Unfavorable undercuts in the bulb portion cast were blocked with clay material, and a modeling wax of 2 mm thickness was used to line the inner wall of the bulb cast (Figure 2).

**Figure 3:**
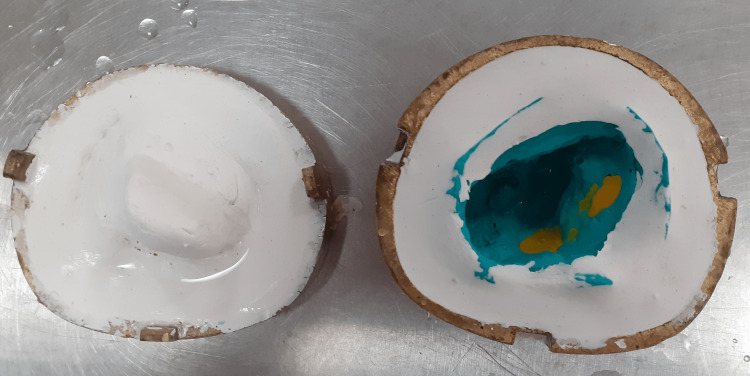
De-waxed hollow part of the obturator

Then, the wax was replaced with heat cure acrylic resin (DPI Heat Cure, Dental Product of India (DPI), Mumbai, India) using routine laboratory procedures (Figure 3).

**Figure 4:**
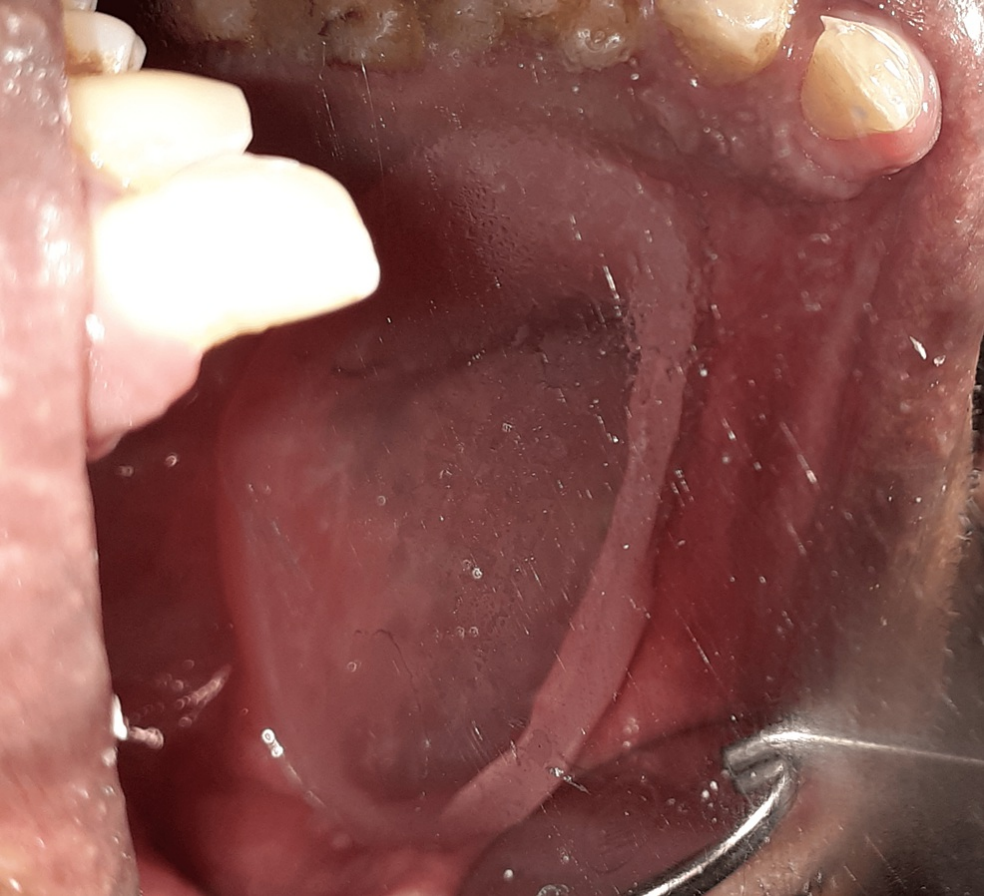
Hollow bulb part of the obturator

After processing, the fit and comfort of the acrylic bulb were checked in the patient’s mouth (Figure 4).

The shellac base plate was used to form the template of the lid covering the open portion of the bulb, and later, it was replaced with heat cure acrylic resin using routine laboratory procedures. Using self-cure acrylic resin, the acrylic lid was attached to the open part of the bulb (DPI RR Cold Cure, DPI, Mumbai, India). The seal between the lid and the bulb was examined to see if there was any water passage between them in order to avoid any contamination in a closed bulb obturator.

**Figure 5:**
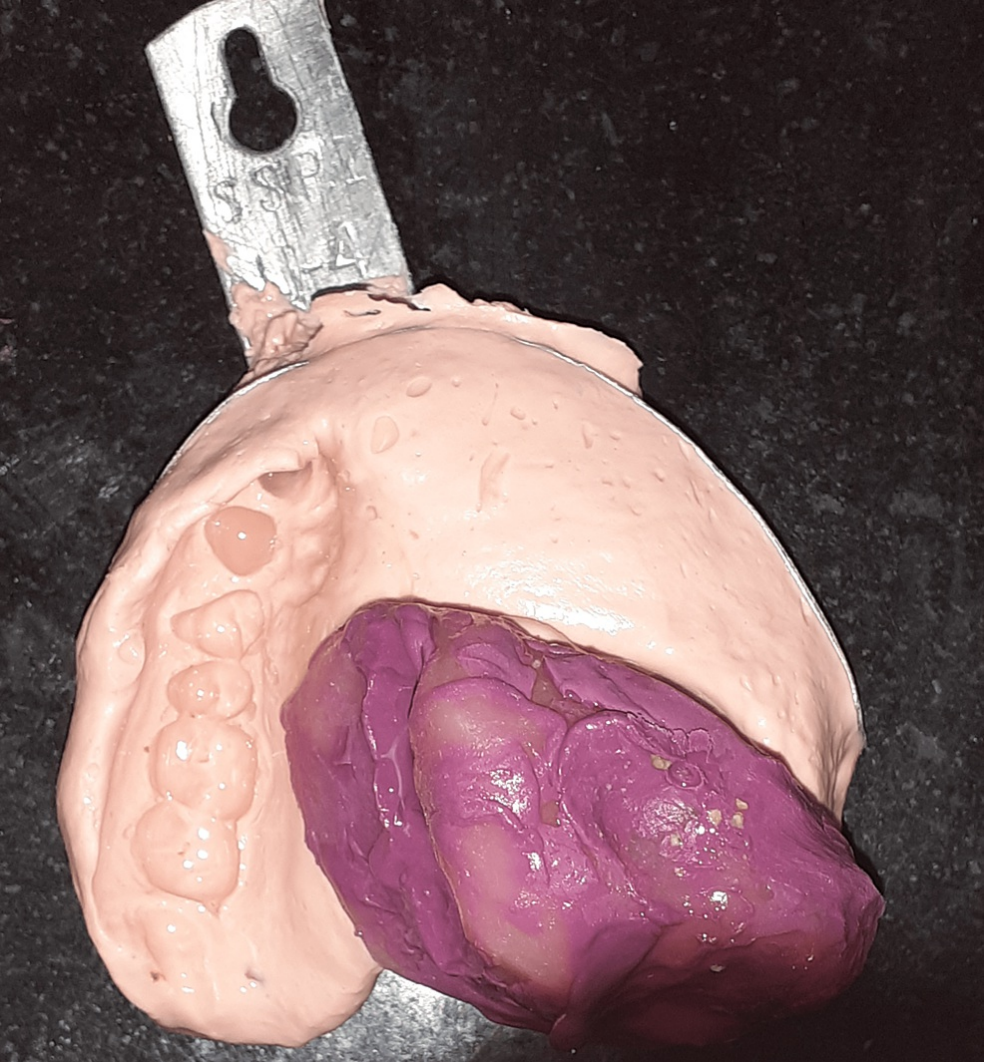
Secondary impression

The hollow bulb was inserted into the maxillary defect, and an impression with alginate (DPI Algitex, DPI, Mumbai, India) was made for the fabrication of the palatal part of the prosthesis. A dental stone was used to pour the cast. To make a custom tray, self-cure resin (DPI RR Cold Cure, DPI, Mumbai, India) was used. The border was molded using a green stick compound (DPI Pinnacle Tracing Sticks, DPI, Mumbai, India), and the master impression of the defect was recorded using a monophase addition silicone impression material (Aquasil, DENTSPLY Sirona, New Delhi, India). With an irreversible hydrocolloid (Zelgan 2002, DENTSPLY, New Delhi, India) and a perforated stock tray, a pick-up impression was made over it (Figure 5).

Die stone was used to pour the master cast (Kalabhai Ultrarock, Vasa Denticity, New Delhi, India). The record base and occlusal rim were fabricated in the master cast, and then, the jaw relation was completed. Semi-anatomic teeth (Radiant, Radiant Surgident, Indore, India) were arranged, and the prosthesis was evaluated for centric occlusion with no contacts in an eccentric position with mandibular teeth, esthetic appearance, and support of underlying tissues.

After the wax try-in, the clasps were fabricated with a 19-gauge wire to aid in additional retention and stability for the prosthesis on the intact teeth. The palatal part of the obturator was fabricated separately. The routine laboratory procedure of flasking, dewaxing, packing, and curing of heat cure acrylic resin was followed. The prosthesis was trimmed and polished, and necessary adjustments were made to fit the prosthesis intraorally.

**Figure 6:**
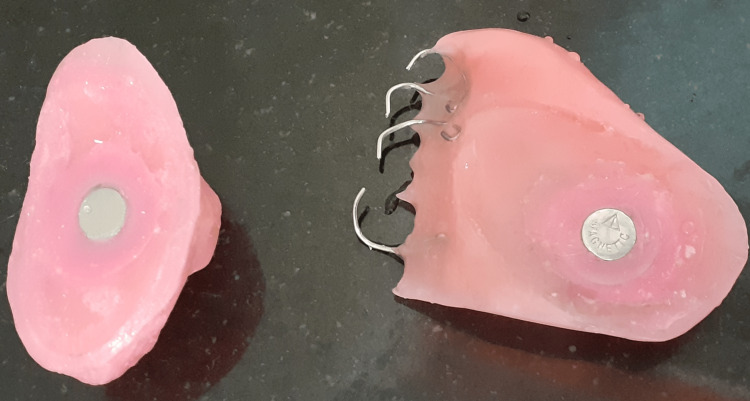
Magnets attached to the obturator

The magnets (cobalt-samarium, Ambica Corporation, New Delhi, India) were used to attach the bulb portion to the palatal part of the obturator (Figure 6).

**Figure 7:**
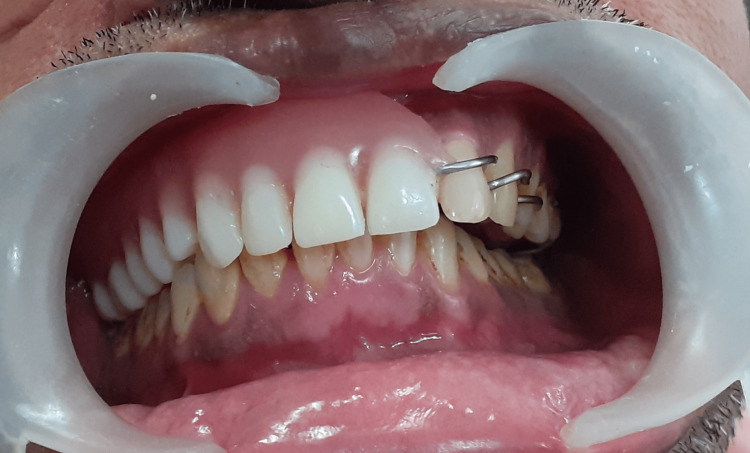
Denture inserted

The magnet is placed within the superior wall of the lid, which covers the antrum and the inferior palatal part of the obturator, with the help of auto-polymerizing acrylic resin. The prosthesis was inserted and evaluated for retention and support (Figure 7).

The patient was taught how to insert the prosthesis: first, the antral part was inserted, followed by the oral part. The patient was also taught to remove the prosthesis, first by removing the oral part while stabilizing the finger on the other side and then by removing the antral part of the prosthesis. The very next day, the patient called for a review. Initially, the patient had difficulties swallowing, and after a month during the review, the patient had generally got accustomed to the prosthesis. The patient was reviewed periodically at regular intervals of three months, six months, and one year. There was no difficulty in speech, mastication, and esthetics, and the patient was able to continue his normal life without any difficulties.

## Discussion

Subtotal maxillectomy has been used as a method of treatment for squamous cell carcinoma in this patient, accounting for 80% of tumors [9]. The removal of a part of the maxilla along with the teeth can lead to difficulty in speech, mastication, and maintenance of proper occlusion [10]. The majority of patients with stage I or II oral cavity cancers respond well to surgery and/or radiation therapy [11]. Another option is chemoradiation, which combines chemotherapy and radiation. Both surgery and radiation are effective treatments for these cancers [12]. Non-vascularized grafts, local-regional flaps, free flaps, and obturator prostheses are all options in reconstructive surgery. Each method has benefits and drawbacks, but the obturator prosthesis enables a successful reconstruction that is partially connected with the extent of the excision of the vertical and horizontal components [13]. To restore functionality, the defect must be adequately closed to keep air, liquid, and food from passing between the nasal and oral cavities. Furthermore, the obturator buttresses the facial tissues in large surgical resections.

A two-piece hollow obturator has the advantage of being easier to use. A hollow bulb obturator allows for the creation of a weightless prosthesis that is considered acceptable by the patient and simultaneously extends into the defective areas. A two-piece obturator is used since the size of the maxillary defect is large, and it has favorable undercuts. A hollow obturator reduces weight and is sterile and simpler to fabricate, which improves speech intelligibility; additionally, a closed hollow obturator intercepts food and fluid accumulation, minimizes air space, and permits maximal extent and comfort than a single-piece obturator [14]. In contrast, an open bulb is inconvenient, noxious, and distressing for the patient [15].

Although there are various techniques used for the construction of hollow bulb obturators, a few techniques such as silicon putty, salt, alum, wax, polyurethane foam, and cellophane-wrapped asbestos are most commonly used [8,16]. The disadvantages of putty are it is difficult to remove after processing, and it is time-consuming [16]. The disadvantages of salt include brittleness and possible displacement during fabrication, it cannot ensure uniform thickness, and it leads to porosity due to cross-reaction with acrylic resin [16]. The disadvantages of alum are failing to remove the cellophane and having trouble molding the alum crystals [16]. The drawbacks of polyurethane foam include its uncertainty, length, complexity in removal after curing, and inability to maintain a uniform thickness in the hollow portion [16]. The disadvantage of cellophane-wrapped asbestos is allergic reaction to asbestos, and it is difficult to remove [16]. The pros of our technique are that the uniform thickness of the wax ensures a uniform thickness of the bulb, there is no need to remove any material after processing, and it has adequate strength and maximum hollowness. The only con is the need to fabricate the lid of the bulb separately, which is acceptable compared to the advantages of this technique.

Magnets have been used since they are small, attractive, and easy to place and fabricate. It has the characteristics of being immune to demagnetization, a strong magnetic field, and a balanced temperature control ranging from good to moderately high. They enhance retention while remaining adaptable during repeated removal and placement [17]. The drawbacks are low corrosion resistance and high cost [18]. Magnets can successfully achieve bilateral retention of extraoral-intraoral prostheses. Breakaway load, which is measured in grams, refers to the power required to detach magnets [18]. It is indirectly proportional to the inclination and the distance between the magnets [18]. These magnets are completely biocompatible and do not have any adverse consequences on the human body [18]. Inert magnetic fields are not harmful to the tissues and are used effectively in patients who have cardiac pacemakers [18]. Coated magnets have been shown to withstand the corrosiveness of saliva, resulting in no harm to oral tissues [19]. However, regular recalls and reviews will be required to ensure the durability of the magnet and the prosthesis. If necessary, chairside replacement of damaged magnets should be done and the prosthesis relined to maintain speech, esthetics, and function [19].

Obturators, which have been used to replace the defect, need retention and stability. This has been achieved with magnets and clasps to dissipate lateral forces, cross-arch stabilization, and retention. Magnets in coin form have more advantages in maxillofacial prosthetics than other forms; the magnet can be selected based on the size of the defect and any diameter that is required [19]. The methodology of treatment should be explained to the patients, along with the pros and cons and the need to replace the magnets if necessary along with a periodic review. A cast denture prosthesis was not fabricated for the patient because of financial constraints, due to which a prosthesis with heat cure auto-polymerizing resin was given.

## Conclusions

A subtotal maxillectomy with an associated large defect was successfully rehabilitated using a hollow, two-component obturator. Acquired diseases such as carcinomas can have far-reaching psychological, functional, and surgical consequences for humans. When the defect is large, restoring function, speech, and esthetics can be difficult due to surgery and radiation therapy. The magnets that should be used in such cases to fabricate a multicomponent prosthesis must be specific to hit the target in terms of the patient’s rehabilitation success.
